# Supplementary material for: Theoretical Study on Symmetry-Broken Plasmonic Optical Tweezers for Heterogeneous Noble-Metal-Based Nano-Bowtie Antennas
Source: Nanomaterials (Basel). 2021 Mar 17;11(3):759. doi: 10.3390/nano11030759 (PMC8002932; doi:10.3390/nano11030759)
Supplement: Supplementary file 1 [file nanomaterials-11-00759-s001.pdf]

nanomaterials

Supplementary material

Theoretical Study on Symmetry-broken Plasmonic Optical Tweezers for Heterogeneous Noble Metal-based Nano-bowtie Antennas

Guangqing Du, Yu Lu, Dayantha Lankanath, Xun Hou and Feng Chen *

State Key Laboratory for Manufacturing System Engineering and Shaanxi Key Laboratory of Photonics Technology for Information, School of Electronic Science and Engineering, Xi'an Jiaotong University, Xi'an, 710049, China; guangqingdu@mail.xjtu.edu.cn (G.D); zjkly19900714@126.com (Y.L.); dyanthalankanath1@gmail.com (D.L.); houxun@mail.xjtu.edu.cn (X.H.)

* Correspondence: chenfeng@mail.xjtu.edu.cn

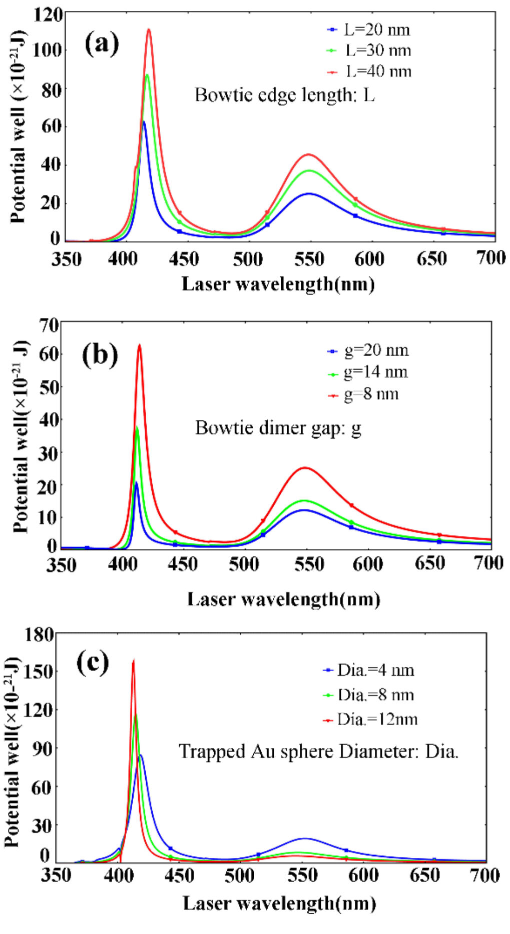

Figure S1. The plasmonic potential well vs. laser wavelength with respect to different bowtie edge length in Figure S1 (**a**); bowtie gap in Figure S1 (**b**); trapped Au sphere diameter in Figure S1(**c**). The fixed bowtie gap taken as 8nm in Figure S1(**a**); the bowtie edge length is set as 20nm in Figure S1 (**b**); the bowtie gap is taken as 20nm in Figure S1(**c**). We can see that the potential well at the working wavelength (spectrum peak point) can be significantly enhanced as enlarging the bowtie edge length from 20nm to 40nm, as in Figure S1(**a**), or increasing the trapped Au sphere diameter from 4nm to 12nm shown in Figure S1(**c**), which is considered a size comparable to the typical quantum dots. However, the potential well can be lowered for a decreased bowtie gap from 20nm to 8nm, as in Figure S1 (**b**).

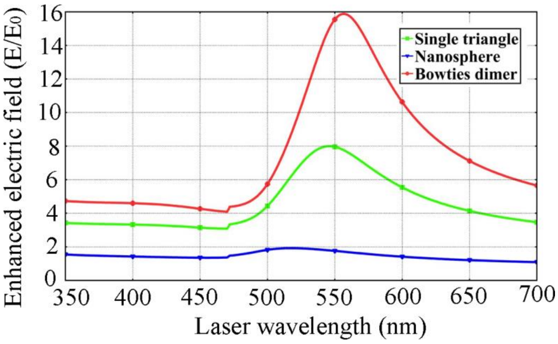

Figure S2. The calculated enhanced electric field for understanding of the optical properties of Au nanosphere, single Au triangle, and Au-Au bowties dimer. We set the geometrical edge length for the triangle to 40nm. It can be seen that the enhanced electric field can be significantly increased for the bowtie dimer compared to the geometries of single triangle at the wavelength 560 nm. The nanosphere size is comparatively taken as the equal one with the single triangle here. As a result, the large trapping potential and stable optical trapping can be expectedly generated via utilizing the bowties dimer instead of single plasmon nano-geometry.
